# Development and validation of a novel predictive model and web calculator for evaluating transfusion risk after spinal fusion for spinal tuberculosis: a retrospective cohort study

**DOI:** 10.1186/s12891-021-04715-6

**Published:** 2021-09-25

**Authors:** Shengtao Dong, Wenle Li, Zhi-Ri Tang, Haosheng Wang, Hao Pei, Bo Yuan

**Affiliations:** 1 Department of Spine Surgery, Second Affiliated Hospital of Dalian Medical University, Dalian, 116021 China; 2 Department of Orthopedics, Xianyang Central Hospital, Xianyang, 712000 China; 3 School of Physics and Technology, Wuhan University, Wuhan, 430072 China; 4 Department of Orthopaedics, Second Hospital of Jilin University, Changchun, 130000 China; 5 Department of Orthopaedic Trauma, Second Affiliated Hospital of Dalian Medical University, Dalian, 116021 China; 6 Department of Reparative and Reconstructive Surgery, Second Affiliated Hospital of Dalian Medical University, Dalian, 116021 China

**Keywords:** Blood transfusion, Spinal tuberculosis, Spinal fusion, Machine learning, Prediction model, Shiny application

## Abstract

**Objectives:**

The incidence and adverse events of postoperative blood transfusion in spinal tuberculosis (TB) have attracted increasing attention. Our purpose was to develop a prediction model to evaluate blood transfusion risk after spinal fusion (SF) for spinal TB.

**Methods:**

Nomogram and machine learning algorithms, support vector machine (SVM), decision tree (DT), multilayer perceptron (MLP), Naive Bayesian (NB), k-nearest neighbors (K-NN) and random forest (RF), were constructed to identified predictors of blood transfusion from all spinal TB cases treated by SF in our department between May 2010 and April 2020. The prediction performance of the models was evaluated by 10-fold cross-validation. We calculated the average AUC and the maximum AUC, then demonstrated the ROC curve with maximum AUC.

**Results:**

The collected cohort ultimately was consisted of 152 patients, where 56 required allogeneic blood transfusions. The predictors were surgical duration, preoperative Hb, preoperative ABL, preoperative MCHC, number of fused vertebrae, IBL, and anticoagulant history. We obtained the average AUC of nomogram (0.75), SVM (0.62), k-NM (0.65), DT (0.56), NB (0.74), MLP (0.56) and RF (0.72). An interactive web calculator based on this model has been provided (https://drwenleli.shinyapps.io/STTapp/).

**Conclusions:**

We confirmed seven independent risk factors affecting blood transfusion and diagramed them with the nomogram and web calculator.

## Introduction

The incidence with regard to tuberculosis was approximately a quarter of global population and the *Mycobacterium tuberculosis* causes 1.5 million mortalities annually [1]. Although developing countries, particularly southern Africa, China and India, account for more than half of all cases, unfortunately, emerging immunodeficiency and anti-tuberculosis drugs resistance place a burden on TB treatment in western countries [2]. In TB patients, anemia is a frequent comorbidity with estimated prevalence more than one third [3]. The causes of TB patients with anemia are variable, which mainly include iron-deficiency anemia and anemia of inflammation or both. The conclusion of Minchella et al. [3] and Gil-santana et al. [4] identified that inflammation is a more plausible explanation for TB-associated anemia.

Skeletal TB is diagnosed in nearly 10% of all TB cases, and spine is the most commonly involved site representing 50% of these cases [5]. With its insidious onset and chronic progression, patients initially present with back pain and limited vertebral motion in addition to symptoms of TB systemic toxicity. Complications such as clod abscess, kyphosis and neurological deficits may arise if diagnosis and treatment are delayed. Late-onset paraplegia is the most devastating clinical presentation, occurring in 10 to 30% of patients with spinal TB [2, 6].

With the advancement of anti-tuberculosis drugs, modern surgical purpose is primarily to debride lesion, furthermore to decompress the spinal cord and nerves and reestablish spinal stability. Because of the superiority of spinal fusion (SF) in stabilizing the vertebra, this approach is widely used in many spinal diseases [7]. However, it is necessary to be aware the significant blood loss in the perioperative period of SF including estimated blood loss (EBL) and hidden blood loss (HBL) [8, 9]. In particular, according to the high incidence of anemia in TB patients, blood transfusion seems to be an inescapable problem for these patients.

Artificial intelligence (AI) is leading the medical revolution, and the performance of machine learning (ML) in deep mining of data is satisfactory. These algorithms learn autonomously and can train to recognize patterns faster and more accurately than humans when researchers provide them with high-latitude data [10]. As previously reported, machine learning has been widely incorporated into tuberculosis research [11]. Chen and his colleagues [12] have also used machine learning algorithms such as multilayer perceptrons to perform detailed analysis of the genotypes of multidrug resistance Mycobacterium tuberculosis, which has greatly facilitated the clinical management of tuberculosis.

In the present study, we developed a traditional statistical algorithm and machine learning algorithms to assess transfusion risk factors in patients with spinal tuberculosis who underwent spinal fusion. This human-computer interactive predictive model provides a great predictive accuracy. The optimal prediction model obtained from the validation is used to build an online calculator.

## Methods

### Patients and data collection

In this population-based retrospective cohort study, we gathered all patients who presented to the department of spine surgery between May 2010 and April 2020 and were clinically diagnosed with spinal TB. The inclusion criteria: 1. Patients diagnosed with spinal tuberculosis; 2. Patients who underwent SF; 3. The collected data is complete and available. The exclusion criteria: 1. Hematological diseases, severe liver disease, chronic kidney disease and malignant tumor; 2. Brucellosis; 3. Disc herniation; 4. Spinal stenosis; 5. Spondylolisthesis; 6. Scoliosis deformity; 7. Vertebral fracture and dislocation; 8. Revision surgery; 9. Emergency surgery; 10. Minimally invasive fusion surgery; 11. Pre-deposit autologous blood transfusion; 12. Preoperative blood transfusion; 13. Despite the indication for blood transfusion, the patient and family refused the transfusion. The diagnosis of spinal tuberculosis is based on clinical history, imaging, laboratory tests and tissue culture.1) Clinical symptoms include constitutional symptoms of tuberculosis, cold abscesses, lymphadenopathy, back pain, neurologic deficits, and spinal deformity; 2) Imaging revealed multiple involved vertebral bodies and well-preserved discs as well as soft tissue shadowing due to cold abscesses; 3) Laboratory test Non-specific alterations (erythrocyte sedimentation rate, C-reactive protein and leukocytes) and specific result (tuberculosis skin test) obtained by laboratory tests; 4) Histological diagnosis including culture, histopathology and, if necessary, polymorphic enzyme chain reaction.

Patient demographic characteristics, drug history, surgical factors and clinical parameters were collected to identify potential risk factors, including age, sex, comorbid diseases (e.g., hypertension and diabetes), anticoagulant history, paraplegia, length of hospitalization, time to definitive surgery, surgical duration, intraoperative blood loss (IBL), number of fused vertebrae, preoperative laboratory indicators, and expenses of hospitalization and blood transfusion. All subject data were obtained from our electronic medical records, and the study protocol was approved by our institutional review board. R studio and Python are used to produce figures.

**Fig. 1:**
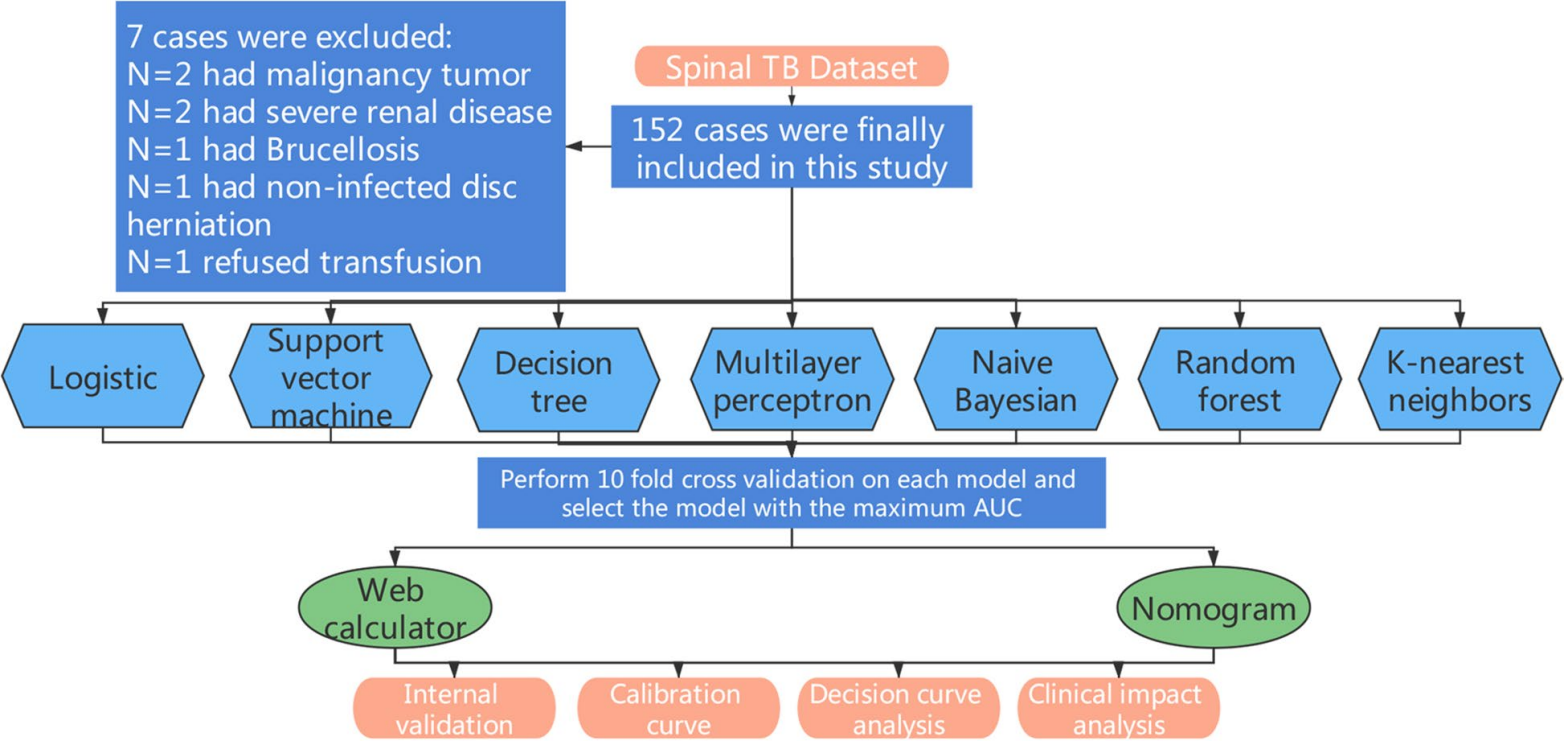
The flowchart of this study

A systematic review performed by Carson et al. [13] indicated that a restrictive transfusion strategy (Hb level of 70–90 g/L) contributed to reduce transfusion rates by 39–43% and immunize mortality, complication rates and readmission rates within 30 days postoperatively against unrestricted transfusion strategy. Therefore, our institution adheres to the following measures: blood transfusion requirements were permitted if the patient has an Hb < 70 g/L or an Hb of 70-100 g/L in cases of advanced age and poor cardiopulmonary function. We adopted autologous transfusion techniques and calculate the IBL by the difference between the total suction volume and the total flush volume. We defined anticoagulant history as heparin, low-molecular heparin or warfarin taken within seven days before surgery. Preoperative laboratory indicators included PT, APTT, FBG, Hb, WBC, PLT, HCT, MCV, MCH, MCHC, RDWCV, ALB, ESR, CPR. The flowchart of the study was presented in Fig. 1.

### Statistical analysis

All data statistical analysis were performed using SPSS version 22.0 (IBM SPSS, Armonk, New York). Difference in continuous data and categorical data were analyzed using the independent sample t-test and chi-square test, and were described with mean and standard deviation (SD), and percentages, respectively. Univariate and multivariate logistic regression were used to identify the risk of blood transfusion after spinal fusion. Statistical significance level was defined as *p* < 0.05.

### Development of the nomogram

All independent risk factors (*p* < 0.05) determined by multivariate regression were entered into the nomogram. Our prediction model graphically presented all significant associations of blood transfusion in patients with spinal TB undergoing SF.

### Support vector machine

Support vector machine (SVM) is a supervised classification model [14]. We used SVM with the linear kernel to classify the patients via a recursive feature elimination approach. The implementation of the code depends on the programming language Python 3.8 and data mining algorithm Scikit-Learn 0.22. The accuracy of SVM is the criterion to evaluate the classification performance after training.

### Decision tree

Decision tree (DT) conform to a tree classification scheme, where nodes symbolize input variables and leaves symbolize decision outcomes [15].

### Multilayer perceptron

Multilayer perceptron (MLP) is a feedforward neural network, which can fit high-dimensional data. Except the input layer, each neuron in other layers has a nonlinear activation function [16].

### Naive Bayesian (NB)

Naive Bayesian models are a common binary classifier in machine learning. The theory basis of NB is the Bayesian rule, which aims to give the probability of a sample belonging to each category [17].

### K-nearest neighbors

K-nearest neighbors (K-NN) classifies unlabeled observations based on a similarity measure (e.g., distance function), which aims to find the k samples that are closest to the training sample [18].

### Ensemble learning

To further verify the performance of machine learning in this work, an ensemble learning method, random forest (RF), is selected. As an ensemble learning method, RF gives the classification results based on the class selected by the most trees, which can be also regarded as a voting ensemble method.

### Parameter selection

Specifically, the linear kernel function is selected in SVM with parameter C equalling to 1. For MLP, the number of layers and neurons in the hidden layer are set as 3 and 5, respectively. The assumption distribution of NB is set as Gaussian distribution. The maximum depth of DT is set as 4 and the number of estimators in RF is set as 10. In KNN, the value of k is set as 3.

### Model capability validation and evaluation

To determine which model was superior in predicting blood transfusion from the multidimensional data, the obtained 10-fold cross-validation results were compared. The average of the area under the ROC curve (AUC) for 10-fold cross-validation represents the predictive power of the model, and the ROC curve is plotted according to the maximum AUC. The threshold value of the model prediction ability is AUC > 0.5. A larger AUC corresponds to a stronger prediction performance. Meanwhile, we took additional measures to evaluate nomogram, including decision analysis curves, calibration plots and clinical impact curve.

### Shiny web calculator

To improve the feasibility of using this predictive model for clinicians, we have designed a web calculator using shiny package 1.3.2. This shiny application was uploaded to Shinyapps.io in order to make it freely available to all users.

## Result

### Baseline information of patients

A total of 152 patients were enrolled in the study after excluding 7 cases (2 with malignancy tumor, 2 with severe renal disease, 1 with Brucellosis, 1 with non-infected disc herniation, and 1 refused transfusion) and 56 patients (36.8%) required allogeneic blood transfusions after SF. Details of patients’ demographics and baseline characteristics are recorded Table 1. Statistical significance was found in the parameters listed below: anticoagulant history(*p* = 0.020), paraplegia(*p* = 0.022), surgical duration(*p* = 0.009), IBL(*p* = 0.018), number of fused vertebrae(*p* = 0.044), preoperative Hb(*p* = 0.018), preoperative MCHC(*p* = 0.044), preoperative ALB(*p* = 0.030), hospitalization expenses(*p* < 0.001). Higher anticoagulants, paraplegia, length of hospitalization, surgical duration, IBL, number of fused vertebrae and hospitalization expenses in transfusion group, whereas higher preoperative Hb, MCHC and ALB in non-transfusion cohort.

**Table 1:** Demographics and Baseline Characteristics of All Patients

Variables	Non-transfused cohort	Transfused cohort	***P*** value
Number of patients	96	56	
Age (years)	53.52 ± 17.80 (23, 83)	54.98 ± 17.41 (13, 84)	0.623
Sex (male/female)	50/46	31/25	0.696
Hypertension	40 (41.67%)	27 (48.21%)	0.057
Diabetes	15 (15.63%)	11 (19.64%)	0.526
Anticoagulant history	14 (14.58%)	17 (30%)	0.020
Paraplegia	0	3 (5.36%)	0.022
Length of hospitalization (days)	19.63 ± 5.98 (11, 34)	21.55 ± 5.16 (13, 37)	0.047
Time to definitive surgery (days)	12.29 ± 9.19 (2, 42)	12.86 ± 9.20 (2, 39)	0.715
Surgical duration (min)	214.38 ± 66.36 (95, 410)	243.27 ± 61.92 (95, 415)	0.009
IBL (ml)	596.98 ± 556.90 (100, 2500)	811.25 ± 493.22 (250, 3400)	0.018
Number of fused vertebrae			0.044
1	42 (43.75%)	19 (33.93%)	
2	30 (31.25%)	12 (21.43%)	
≥3	24 (25.00%)	25 (44.64%)	
P-PT (s)	12.37 ± 1.00 (10.30, 14.80)	12.36 ± 0.95 (10.90, 14.80)	0.949
P-APTT (s)	31.42 ± 4.79 (23.70, 41.50)	32.05 ± 5.56 (24.10, 52.00)	0.463
P-FBG (g/L)	3.39 ± 1.20 (0.71, 5.63)	3.24 ± 1.22 (0.82, 5.77)	0.472
P-Hb (g/L)	111.01 ± 11.06 (85, 133)	106.80 ± 9.37 (89, 132)	0.018
P-WBC (10^9/L)	6.64 ± 2.13 (2.69, 14.69)	6.80 ± 2.28 (3.12, 15.21)	0.680
P-PLT (10^9/L)	249.07 ± 52.81 (159, 363)	251.02 ± 78.38 (113, 413)	0.855
P-HCT (%)	35.17 ± 3.64 (25.2, 40.8)	34.13 ± 3.79 (24.0, 39.8)	0.096
P-MCV (fl)	86.95 ± 4.01 (79.90, 92.50)	85.98 ± 4.81 (74.50, 97.10)	0.182
P-MCH (pg)	27.59 ± 1.37 (25.5, 30.8)	27.06 ± 2.12 (22.1, 31.6)	0.065
P-MCHC (g/L)	332.75 ± 7.93 (315, 349)	329.68 ± 10.60 (304, 351)	0.044
P-RDWCV (%)	13.48 ± 0.46 (12.60, 14.50)	13.75 ± 1.29 (11.30, 18.00)	0.065
P-ALB (g/L)	38.59 ± 4.04 (29.80, 45.77)	37.02 ± 4.63 (22.25, 45.00)	0.030
P-ESR (mm/h)	33.82 ± 12.72 (11, 81)	37.79 ± 16.40 (8, 73)	0.099
P-CPR (mg/L)	33.93 ± 15.74 (3.00, 74.00)	35.80 ± 15.84 (4.86, 80.00)	0.482
Hospitalization expenses (RMB)	72,635.7 ± 31,245 (8168.86, 115,657.69)	122,083.9 ± 34,483.8 (63,959.12, 201,266.44)	0.000
Blood transfusion expenses (RMB)	0	2498.20 ± 1541.01	

*IBL* Intraoperative blood loss, *P-PT* Preoperative prothrombin time, *P-APTT* Preoperative activated partial thromboplastin time, *P-FBG* Preoperative fibrinogen, *P-Hb* Preoperative hemoglobin, *P-WBC* Preoperative white blood cell, *P-PLT* Preoperative platelet, *P-HCT* Preoperative hematocrit, *P-MCV* Preoperative mean corpuscular volume, *P-MCH* Preoperative mean corpuscular hemoglobin, *P-MCHC* Mean corpuscular hemoglobin concentration, *P-RDWCV* Preoperative red cell volume distribution width coefficient variation, *P-ALB* Preoperative albumin, *P-ESR* Erythrocyte sedimentation rate, *P-CPR* Preoperative C reactive protein.

### Univariate and multifactorial logistic regression analyses

Predictors with *p* value less than 0.05 obtained from the univariate logistic regression analyses were included in the multifactorial logistic regression analyses. The final independent risk factors identified included surgical duration (OR = 1.008, 95%CI 1.002–1.014, *p* = 0.007), IBL (OR = 1.001, 95%CI 1.000–1.002, *p* = 0.012), number of fused vertebrae (OR = 2.034, 95%CI 1.261–3.280, *p* = 0.004), anticoagulant history (OR = 3.281, 95%CI 1.296–8.360, *p* = 0.012), preoperative Hb (OR = 0.954, 95%CI 0.918–0.991, *p* = 0.015), preoperative ALB (OR = 0.908, 95%CI 0.831–0.992, *p* = 0.032), preoperative MCHC (OR = 0.953, 95%CI 0.914–0.993, *p* = 0.022). The specific results are shown (in Table 2).

**Table 2:** Univariate Analysis and Multivariate Analysis Associated with Risk Factors for Blood Transfusion in patients with spinal tuberculosis

Variable	Univariable logistic regression analysis				Multivariable logistic regression analysis			
	OR	95% CI		*P* value	OR	95% CI		*P* value
		Lower	Upper			Lower	Upper	
Surgical duration	1.007	1.002	1.012	0.011	1.008	1.002	1.014	0.007
IBL	1.001	1.000	1.001	0.025	1.001	1.000	1.002	0.012
Number of fused vertebrae	1.513	1.019	2.247	0.040	2.034	1.261	3.280	0.004
Anticoagulant history	2.553	1.143	5.702	0.022	3.281	1.296	8.306	0.012
Preoperative Hb	0.962	0.931	0.994	0.020	0.954	0.918	0.991	0.015
Preoperative ALB	0.918	0.848	0.993	0.033	0.908	0.831	0.992	0.032
Preoperative MCHC	0.949	0.914	0.986	0.008	0.953	0.914	0.993	0.022

*OR* Odds ratio, *95% CI* 95% Confidence interval.

### Evaluation of selected transfusion risk via nomogram and machine learning methods

**Fig. 2:**
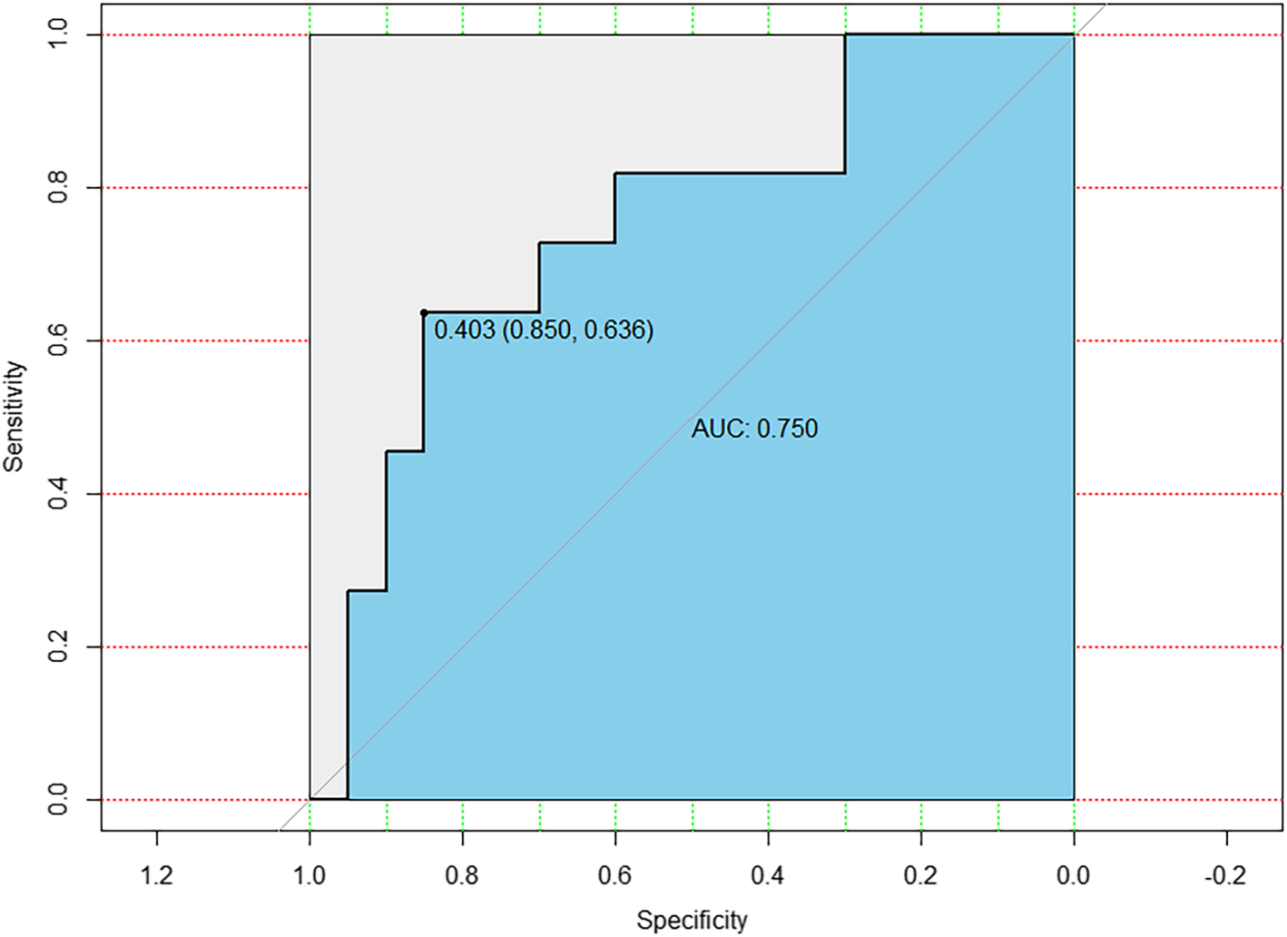
ROC curve for maximum AUC values calculated in 10-fold cross validation from nomogram

**Fig. 3:**
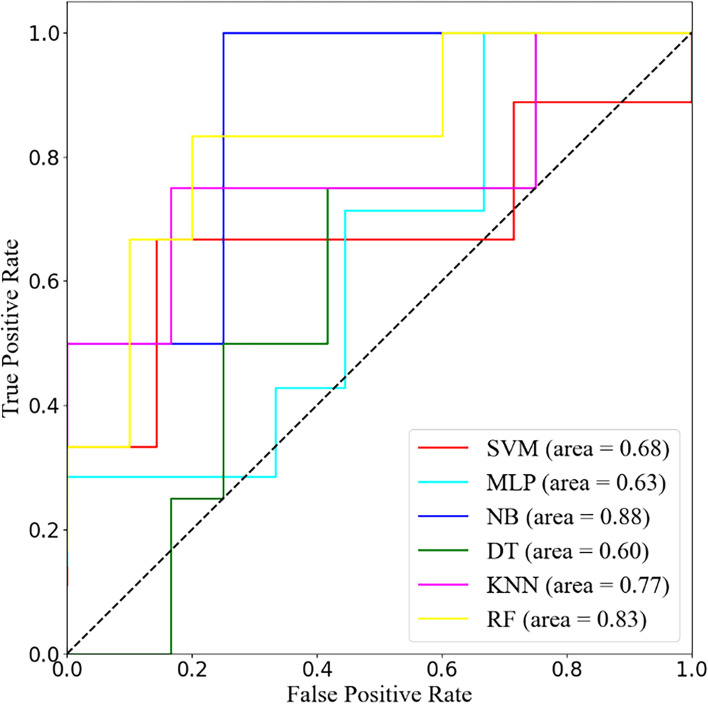
The optimal ROC curve for machine learning

10-fold cross-validation was used to evaluate the prediction ability of nomogram and selected algorithms. The following 10-fold cross-validation ROC curves show the optimal prediction results for nomogram (Fig. 2.) and SVM, K-NM, DT, NB, MLP and RF (Fig. 3.).

### Evaluation of model predictive capability

Table 3 shows the predictive performance of the nomogram and the selected machine learning models, including the average AUC, maximum AUC, and average ACC. The best average and maximum AUC values were found in the nomogram with 0.75 and 0.93, respectively. The predictive power and accuracy of the first place in the machine learning algorithms was determined by the NB.

**Table 3:** Predictive performance of nomogram and machine learning algorithms

Model	Average AUC	Maximum AUC	Average Accuracy
**Nomogram**	0.75	0.93	
**Support vector machine**	0.62	0.68	0.62
**K-nearest neighbors**	0.65	0.77	0.71
**Decision tree**	0.56	0.60	0.64
**Naive Bayesian**	0.74	0.88	0.73
**Multilayer perceptron**	0.56	0.63	0.65
**Random Forest**	0.72	0.83	0.75

### Construction and validation of the nomogram

**Fig. 4:**
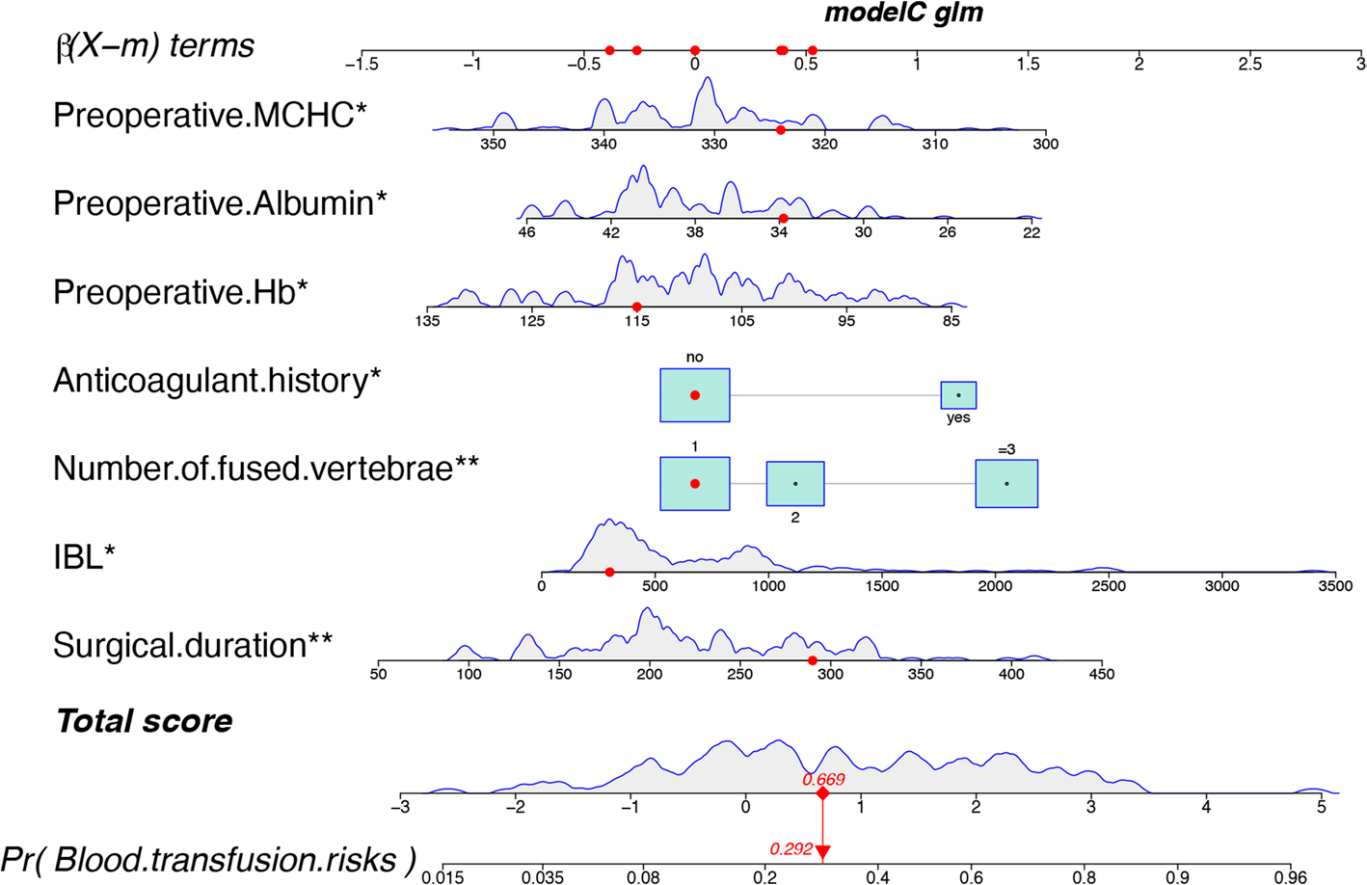
The Nomogram for predicting transfusion risk of spinal TB with spinal fusion

**Fig. 5:**
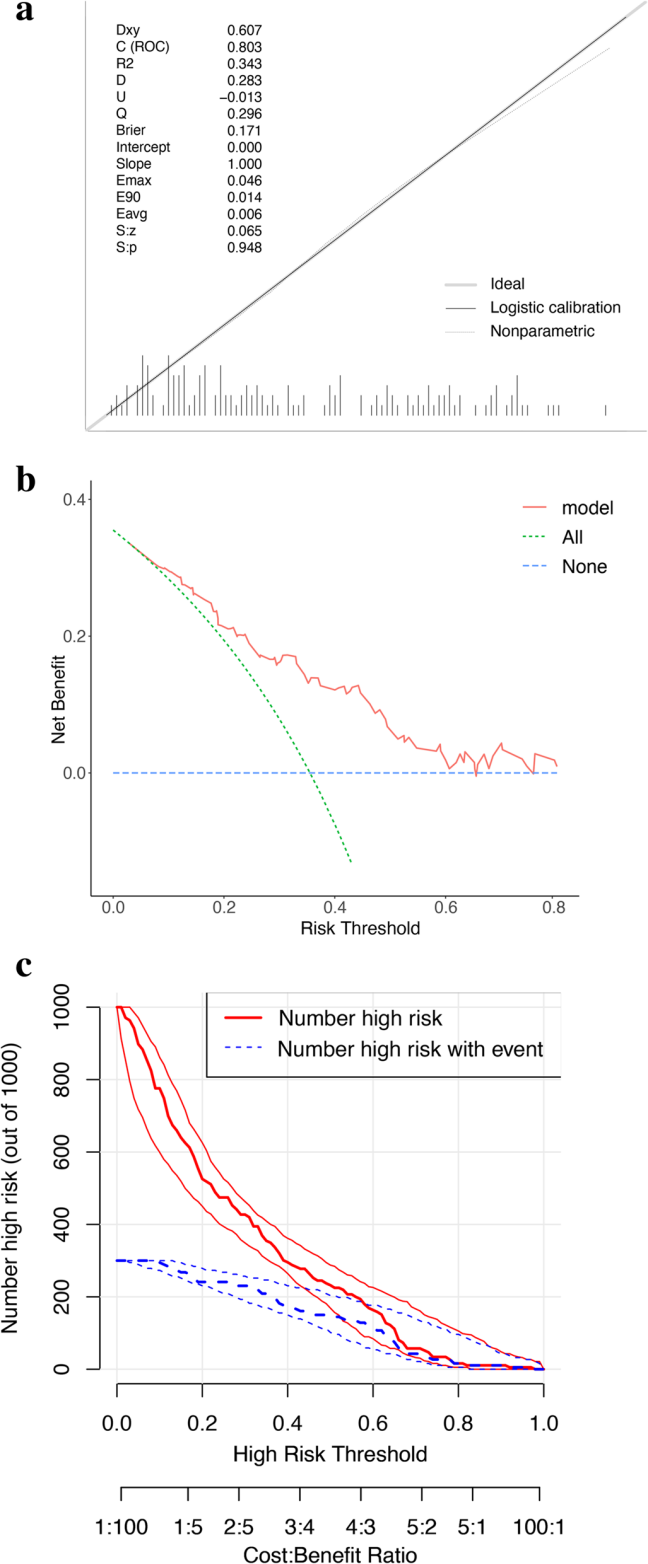
Calibration curve, clinical impact curve and decision curve analysis of blood transfusion risk in patients with spinal tuberculosis. Calibration curve (**a**) and decision curve analysis (**b**) of the training group. Clinical impact analysis (**c**) of Nomogram

Due to the superiority shown by nomogram in 10-fold cross-validation, the statistical results of the logistic regression were developed into a nomogram (Fig. 4). Our nomogram presents the scores of each independent risk factor separately. The scores of the factors were summed, and higher scores suggested higher transfusion risk. The calibration plots of the nomogram confirm the good agreement between the actual and predicted values Fig. 5a. Decision curve analysis demonstrated the qualified clinical utility of our model Fig. 5b. Clinical impact curves Fig. 5c shown a consistent preponderance of predicted high-risk patients within the most favorable threshold probabilities and acceptable cost-effectiveness.

### Web calculator design

In our web calculator, we provide 7 options that can be modified and use “Probability” to represent transfusion risk. After setting each option, clicking on “predict” will get a line in the “Graphical Summary” indicating the risk of blood transfusion. The risks of multiple patients can be displayed simultaneously, which helps to compare risk and develop individual transfusion strategies. Click on the following website to get this calculator: https://drwenleli.shinyapps.io/STTapp/

## Discussion

The gold standard for the diagnosis of spinal TB has reached a consensus that culture confirms the presence of *M.tuberculosis* [6]. However, limited by the low detection rate of mycobacteria in skeleton, diagnosis commonly consisted of clinical symptoms, physical examinations, radiographic manifestations, anti-tuberculosis drugs response, molecular methods, tissue and microbiological assessment, polymerase chain reaction (PCR) and gene detection [5–7]. Due to its disguised clinical presentation and complicated diagnostic targets, vertebral body (VB) destruction was discovered at the patient’s first visit. Previous report demonstrated VB involvement in 98% patients [6]. Antitubercular drugs, the primary approach of managing TB, significantly improves adverse events in these cases, but the surgical intervention of patients with spinal TB cannot be ignored. Notably, patients with spinal TB have lower preoperative hemoglobin [19] and preoperative albumin [20], higher transfusion rates [19–22], longer operative time [21–23], more intraoperative blood loss [23], and more fused vertebrae [23] than patients undergoing SF for other etiologies, suggesting that allogeneic transfusion as a management measure is more frequently used in spinal TB. And the length of hospital stay is thus prolonged [23, 24]. Whereas, the complex intra-human environment in TB patients place them at higher risk for serious transfusion complication [4]. Thus, it is urgent to investigate the risk factors of blood transfusion after SF, develop a convenient and sensitive prediction model, tailor the surgical strategy to the patient’s characteristics, and ensure the optimal functional outcome.

In the current article, there were seven parameters significantly associated with transfusion supported by strong evidences, including preoperative hemoglobin, preoperative albumin, preoperative MCHC, surgical duration, number of fused vertebra and anticoagulation history. As the most readily available test result, we chose the first blood test result after the patient arrived at our department. We reckon that this strategy can minimize heterogeneity and facilitate the model predict the patients’ blood transfusion risk at an early stage.

Preoperative hemoglobin has been verified as a significant predictor of transfusion among published clinical trials conducted in patients undergoing spinal surgeries [21, 25]. In view of the persistent inflammation in TB patients, the balance of iron in the body is disrupted and its absorption is reduced and consumption increased [3]. Therefore, anemia in TB patients is not infrequent and has a complex pathology. In our study, preoperative Hb was 106.80 ± 9.37 in the transfusion set versus 111.01 ± 11.06 in the non-transfusion group. Soliman et al. [19] report that a 49.6-fold increase in the probability of transfusion in patients with preoperative Hb < 11 g/dL compared to those with preoperative Hb > 14 g/dL. We detected that lower preoperative albumin result in higher transfusion requirements (37.02 ± 4.63 vs. 38.59 ± 4.04; *p* = 0.030), which might be related to the poor general condition of TB patients. For patients undergoing surgery for spinal TB, lower preoperative albumin maps out to worsening health status, more complications, longer hospital stays, and possibly increased mortality [26, 27]. Furthermore, low protein-related malnutrition is a risk factor for TB infection and both might be mutually reinforcing [28]. Accordingly, preoperative serum albumin was a potent predictor in evaluating the both of transfusion risk and long-term survival, and initiative preoperative management should be adopted to correct perioperative status and reduce transfusion risk [29]. The potential association between MCHC and blood transfusions appears to be related to the correction of anemia. Some studies have shown that drugs to improve anemia also increase MCHC in patients [30, 31].

As for intraoperative factors, we found that patients that undergone longer surgical duration, more fused vertebrae, and more intraoperative blood loss (IBL) tended to request blood transfusions. The number of fused vertebrae was deemed as the strongest predictor and our speculation is supported by a report from China that it may be the dependent variable affecting the length of surgery and IBL [32]. Multilevel vertebral fusion requires a matching extensive incision and sustained retraction forces to expose the surgical field, which means that a large amount of muscle behind the posterior spine and the posterior complex will hardly be preserved. Medically induced muscle and soft tissue injuries and disruptions in spinal integrity and stability, as well as prolonged surgery times, can increase blood loss during surgery [33]. In the systematic review by Elgafy et al. [34], the threshold for intraoperative blood loss in patients requiring blood transfusion is 650 ml, which is comparable to our report. Morcos et al. [24] found that each additional 60 min of surgery time increases the chance of blood transfusion by 4.2% after a retrospective cohort study of postoperative transfusion risk in posterior fusion.

We found evidence that anticoagulant history be associated with blood transfusion, which was comparable to part of the previous reports. In the literature by Fiasconaro et al. [35], the investigators confirmed the increased odds of cases using aspirin and regular heparin by exploring the potential risk of heparin, low-molecular heparin, and aspirin on blood transfusions. In addition, due to the favorable safety demonstrated by low molecular weight heparin with negligible risk of bleeding and blood transfusion, it is recommended for patients in need [36]. Interestingly, there were contradictions in the patient’s anticoagulant history and routine coagulation test results (PT, APTT and platelet count). The underlying mechanism might be that these measurement methods are not sensitive. Such as PT, this indicator generally serves in evaluating common pathways of procoagulant factors (factors II, V, and X) and significantly reduced tissue factor pathways (factor VII) [37].

Based on the results of logistic regression analysis, we constructed the first predictive model that correlates the risk of blood transfusion with both SF surgery and spinal TB. We acknowledge that nomogram has made a great contribution to clinical decision-making via intuitive visualization and low cost. Both the decision curve analysis and the clinical impact curve indicate the great clinical value of this established nomogram in risk stratification. Furthermore, we found that nomogram is the best prediction model compared to ML with an average AUC of 0.75 and a maximum AUC of 0.93. However, in the digital era, artificial intelligence has penetrated several fields of medicine such as genetics and radiographic and we should not be stagnant [38, 39]. Machine learning techniques has the potential to deliver early and accurate diagnosis, and the ability to evaluate prognosis and develop management among complex clinical datasets. Han et al. [40] and Goyal et al. [41] reported the machine learning algorithms for adverse events and unplanned readmissions associated with SF, respectively. In our modeling, the machine learning algorithms we aggressively selected were support vector machines, decision trees, multilayer perceptron, naive Bayesian, k-nearest neighbors and random forest. The results of five algorithms and nomogram reveal gratifying predictive performance with an average AUC greater than 0.5 for all 10-fold cross-validation.

With the interactive shiny application [42], we created a web calculator that make it easy to compare the effects of different parameters individually and in combination, in order to supply a viable tool for clinical screening of high-risk transfusion patients. (https://drwenleli.shinyapps.io/STTapp/) Based on this platform, we provided users with eight modifiable parameters: surgical duration, IBL, number of fused vertebrae, anticoagulant history, preoperative Hb, preoperative Albumin, preoperative MCHC.

Although we have highlighted the prominent advantages of this constructed model and algorithms, several limitations of present literature are still worthy of attention. First, the small sample size of the collected patients and the fact that they were all from one central institution led to some possible overfitting during the data processing phase. Secondly, there may be bias in our retrospective study. Third, although there are a number of clinical parameters that we use to predict transfusion risk, the interaction between M. tuberculosis and the host remains unclear [43]. Further studies should focus on a broader range of assay metrics and surgical parameters.

## Conclusion

In conclusion, our study achieved the objective of identifying predictors of transfusion in patients with spinal TB undergoing SF and demonstrated multiple predictive models including machine learning algorithms. Although AI is still under development, the broad application prospect of machine learning in clinical big data can be foreseen, and it facilitates the evolution towards precision medicine.

## Data Availability

All data generated or analysed during this study are included in this published article [and its supplementary information files].

## Abbreviations

TB: Tuberculosis
SF: Spinal fusion
ML: Machine learning
SVM: Support vector machine
DT: Decision tree
MLP: Multilayer perceptron
NB: Naive bayesian
K-NN: k-nearest neighbors
RF: Random forest

## Declarations

All methods were carried out in accordance with relevant guidelines and regulations.
